# Toward a Risk-Utility Data Governance Framework for Research Using Genomic and Phenotypic Data in Safe Havens: Multifaceted Review

**DOI:** 10.2196/16346

**Published:** 2020-05-15

**Authors:** Kerina Jones, Helen Daniels, Sharon Heys, Arron Lacey, David V Ford

**Affiliations:** 1 Population Data Science Swansea University Medical School Swansea University Swansea United Kingdom

**Keywords:** genomic data, data safe havens, data governance

## Abstract

**Background:**

Research using genomic data opens up new insights into health and disease. Being able to use the data in association with health and administrative record data held in safe havens can multiply the benefits. However, there is much discussion about the use of genomic data with perceptions of particular challenges in doing so safely and effectively.

**Objective:**

This study aimed to work toward a risk-utility data governance framework for research using genomic and phenotypic data in an anonymized form for research in safe havens.

**Methods:**

We carried out a multifaceted review drawing upon data governance arrangements in published research, case studies of organizations working with genomic and phenotypic data, public views and expectations, and example studies using genomic and phenotypic data in combination. The findings were contextualized against a backdrop of legislative and regulatory requirements and used to create recommendations.

**Results:**

We proposed recommendations toward a risk-utility model with a flexible suite of controls to safeguard privacy and retain data utility for research. These were presented as overarching principles aligned to the core elements in the data sharing framework produced by the Global Alliance for Genomics and Health and as practical control measures distilled from published literature and case studies of operational safe havens to be applied as required at a project-specific level.

**Conclusions:**

The recommendations presented can be used to contribute toward a proportionate data governance framework to promote the safe, socially acceptable use of genomic and phenotypic data in safe havens. They do not purport to eradicate risk but propose case-by-case assessment with transparency and accountability. If the risks are adequately understood and mitigated, there should be no reason that linked genomic and phenotypic data should not be used in an anonymized form for research in safe havens.

## Introduction

### Background

The use of genomic data to revolutionize health research and clinical care is a major expanding area of investigation and development. Research using genomic data opens up new insights into health and disease to inform population health [1] and to develop precision medicine, namely, treatment based on a person’s biological constitution, lifestyle, and environment [2]. Being able to use genomic data in association with health and administrative record data can multiply the benefits by including information such as comorbidities, medication histories, laboratory tests, education records, social issues, and lifestyle factors. This creates a more rounded and realistic picture to avoid genetic determinism and to ground phenotypically observed phenomena. Genomic data can be defined as the totality of a person’s DNA sequence, and genetic data can be defined as the parts of the DNA that code for genes [3]. In this paper, we will refer to genomic data unless we are specifically mentioning genetics. We will refer to health and administrative data as phenotypic data for convenience, while acknowledging that not all the information in these records is ultimately or attributably genomic.

By their nature, genomic data are commonly considered to be among the more sensitive types of personal data for various reasons. These include their persistence through a person’s life (barring a degree of plasticity), their role in predicting disease onset or likelihood, stigmatization for insurance or employment, and impact on kin [4,5]. This has led some to the concept of *genetic exceptionalism*, proposing that genetic data should be subject to new, particularly stringent use restrictions, whereas others argue that, although careful control measures are needed, the fundamental issues are largely the same as for other health data [6]. At the other end of the perception scale, some believe that all human genome sequence arising from publicly funded research should be freely available in the public domain [2]. Crucially, the ability of genomic data to provide in-depth information about a person is the defining factor in their added value for research and clinical care. Debates continue, and we have a conundrum in terms of how to progress the use of genomic data for public benefit while safeguarding individuals from harm.

We believe that there is a need for guidance toward a risk-utility data governance framework to simultaneously mitigate disclosure risk and retain maximum data utility. This is because of the nature of the data and ongoing discussions about the ability to truly anonymize genomic data. Some of this debate arises from a conflation of unique versus identifiable data, which we will return to later in this paper. In the absence of absolute certainty about the anonymization of genomic data beyond a shadow of a doubt, we propose that the framework needs to be practical, enabling research while safeguarding against all manageable risks. In this endeavor, privacy protectionism must be avoided, that is, the application of superfluous control measures that do not enhance privacy but do damage data usefulness [7]. Commonly used nonperturbative disclosure controls include suppression of variables or entire records, aggregation (such as age into bands), and masking (such as of clinic identifiers to protect professional reputation). Further methods include data perturbation (such as variable swapping between records), homomorphic encryption (a technique enabling computations on encrypted data), and other privacy-enhancing technologies [8]. Many of these are still in developmental stages and might not ultimately prove suitable for real-world application without reducing data utility. Even when algorithms are brought to encrypted data, they might not be immune to reidentification risk [9,10].

In a previous study, we explored the views of the general public on access models for genomic data in conjunction with health data [11]. Because so far genomic analyses have not yet revolutionized medical care and the majority of testing in clinical routine is still conventional, the study focused on genomic data that had been primarily collected for research. The 3 models of access presented were open (such as via a website), released externally to approved researchers, and within a data safe haven. We refer to a data safe haven as an infrastructure enabling data access within a secure environment subject to procedural, technical, and physical controls, in combination with disclosure controls applied to the data, to safeguard data subject privacy [12]. Although some people were comfortable with data being open access and more with release to named researchers, the overall preference was for access via data safe havens [11]. It can be argued that dangers differ between researcher-hosted data being unintentionally released externally leading to disclosure, compared with data hosted in a safe haven being reengineered to reveal personal data, as the latter is generally perceived to be more computationally intensive. Even so, because genomic data (largely) persist over the course of a lifetime and are passed to the offspring, it is important to remember that what is impossible today may be achievable within minutes in the not-too-distant future. Nonetheless, at this point in time, we acknowledge the appropriateness of all 3 models in their place and ongoing work to strengthen governance arrangements, for example, in Web-based access to large-scale genomic sequence for genome-wide associations and similar studies [13].

### Objective

Many countries worldwide are investing in infrastructures that enable extensive, population phenotypic data to be accessed in an anonymized form via a data safe haven, and some are also incorporating genomic data (in various forms) to add to the research potential [14-16]. Being able to link data at the individual level while safeguarding privacy is an essential part of these enterprises, enabling information from multiple disparate datasets to be used in research. However, because of the nature of, and perceptions around, genomic data, there are challenges to be overcome to incorporate genomic data in a lawful, ethical, and socially acceptable way. Consequently, the aim of this paper was to work toward a risk-utility data governance framework for using genomic data in conjunction with phenotypic data in an anonymized form for research in data safe havens. We propose that the findings of this study will have particular value in developing guidelines for safe havens and augmenting existing operating models and will also be relevant to some extent for other data access models.

## Methods

We took a multifaceted approach drawing upon data governance arrangements in published research papers, case studies of organizations working with genomic and phenotypic data, public views and expectations for the use of genomic data, and example studies using genomic and phenotypic data. The findings were contextualized against a backdrop of legislative and regulatory requirements and used to create a set of recommendations to inform a risk-utility data governance framework for using genomic and phenotypic data in safe havens. We set out our considerations of these elements in the Results section to draw together the findings to inform the recommendations. Ethical approval was not required for this study because the engagement with members of the public as research participants took place in our previous study [11].

### Legislation and Literature Review

We carried out a summary (nonexhaustive) review of the legislative and regulatory backdrop to gain an insight on issues associated with the use of genomic data in principle. For this, we focused on the European Union (EU) because wider consideration was beyond the scope of this paper and would warrant a separate study. The elements considered were the European Convention on Human Rights and Biomedicine [17] and the General Data Protection Regulation (GDPR) [18] of 2016, augmented by national legislation and official guidance, such as the UK Data Protection Act of 2018 [19] and the work of the UK Information Commissioner’s Office. We appreciate that legislation in other jurisdictions will vary, but we use the ones we have mentioned here as illustrations. In addition to compliance with data protection legislation, there are ethical issues to consider and address. The Global Alliance for Genomics and Health (GA4GH) has several working groups focusing on particular issues and has produced a high-level framework for the use of genomic and health data [20] to be implemented in line with jurisdictional requirements.

We used our previously conducted literature review on uses of genomic and routinely collected phenotypic data to guide this study (Daniels H et al, unpublished data, 2019). As part of the review, we drew out various pieces of information to categorize the studies and the details of data governance arrangements to the extent that they were presented in the publication. The relevant variables were genomic data and source, phenotypic data and source, data access model, and details of data governance. This information enabled us to create examples of data use to consider relative risks for data access via a safe haven. We did this using factors including the format of genomic data being used, the type of health condition being studied, and the extent of data linkage. We note that many of the published studies used an external data release model, but we used them in this context simply as examples of research using genomic and phenotypic data. We also drew upon a series of interviews we had conducted with representatives from safe haven enterprises in Germany, Australia, the United Kingdom, and Canada to add depth to the information in published studies. The relevant questions were on types of genomic data integrated with phenotypic data, main governance challenges encountered and how they were addressed, main access model, and access conditions [21].

### Public Views

Recognizing that public engagement on the use of health data and specifically genomic data is an active area of investigation [22], when we carried out public engagement on the use of genomic and health data, we focused specifically on access models, as noted above [11]. The public engagement activities took the form of 8 workshops with a total of 116 people. As well as taking part in free-form discussions, the participants completed an anonymous exit questionnaire to provide their personal views on the relative benefits and risks of each access model: open, external release, or data safe haven. This had previously not been explored and provided new insights into public preferences to inform this paper.

## Results

### Legislative and Regulatory Backdrop

The EU has expressly recognized the use of genetic information in the European Convention on Human Rights and Biomedicine (1997), which provides for the misuse of biological and medical advances and sets out prohibitions in respect of bioethics and the right to a private life [17]. It further bans decisions made on the basis of genetic characteristics and governs predictive genetic tests for medicinal purposes. Personal data processing is largely governed by the GDPR [18] of 2016, augmented by national legislation and official guidance, such as the UK Data Protection Act of 2018 [19] and the work of the Information Commissioner’s Office. Personal data are defined under Article 4 of the GDPR as “any information relating to an identified or identifiable natural person who can be identified, directly or indirectly in particular by reference to an identifier such as name...or to one or more factors specific to the physical, physiological, genetic specific to that natural person*.*” Recital 34 of the GDPR defines genetic data as “personal data relating to the inherited or acquired genetic characteristics of a natural person which result from the analysis of a biological sample from the natural person in question, in particular chromosomal, deoxyribonucleic acid (DNA) or ribonucleic acid (RNA) analysis or from the analysis of another element enabling equivalent information to be obtained*.*” Processing personal data related to the health of an individual is provided for by the explicit provisions of GDPR Article 9. This provides differing lawful bases for processing and covers a range of data that are considered to be sensitive. Genetic data are expressly included within Article 9.

The key point in applying data protection legislation is that it applies to identifiable personal data from living individuals and not to data from which a person cannot be identified. Although the majority of the genome is identical in all humans, sequence variants occur across the genome that can be used to characterize people and groups. That is not to say that they can be used to identify an individual, as, although the information may be unique, this does not necessarily render it legally identifiable. Within the remit of the GDPR, data from which an individual cannot be identified are legally anonymous. However, identifiers as defined by law do not have to be direct and can be formed from a number of pieces of information. As with any data source, genetic data linked to other data may give rise to a greater risk of identity disclosure, such that it is important to consider whether the combination of available data could lead to the identification of an individual. For example, Y-chromosome repeats were mined from the 1000 Genomes database and could identify people by cross-referencing them with ancestry databases [20]. As far as the GDPR is concerned, it is not correct to state that genetic data are always personal data or will always carry a high risk of disclosure. A proper legal approach would be to assess each use case with expert input to adequately understand the risk of identifiability. Applying legal governance to genetic data can be perceived as challenging because of the esoteric nature of the data, which, furthermore, can be shrouded in scientific terminology. This can appear to complicate the legal issues; however, in essence, data protection law governing genetic data is largely the same as for other health data types.

The GA4GH framework for the use of genomic and health data [21] is to be implemented in line with jurisdictional requirements. The framework establishes a set of foundational principles, namely, to respect individuals, families, and communities; advance research and scientific knowledge; promote health, well-being, and the fair distribution of benefits; and foster trust, integrity, and reciprocity. Furthermore, it proposes core elements of responsible data sharing: transparency; accountability; engagement; data quality and security; privacy, data protection, and confidentiality; risk-benefit analysis; recognition and attribution; sustainability; education and training; and accessibility and dissemination. It does not relate specifically to data safe havens and is broader in scope than our aim to work toward a risk-utility data governance framework for research using genomic data in conjunction with phenotypic data for research in safe havens. However, this valuable document formed a part of the backdrop and, along with the legislation, informed the development of the recommendations.

### Forms of Genomic Data

The literature review on the use of genomic data with phenotypic data revealed a plethora of studies on many health conditions and using various forms of genomic data (Daniels H et al, unpublished data, 2019). Genome-wide association studies (GWAS) and phenome-wide association studies (PheWAS) use statistical methods applied to genomic sequence data to explore and correlate variants and phenotypic traits [22]. However, further processing often takes place so that data derivatives, or metadata, can be taken forward for use with phenotypic data for research or to inform clinical care. This is important when considering data governance issues because not all genomic data used in research are composed of sequence. In fact, the resulting metadata can take many forms of varying complexity. Some examples are as follows: Binary Alignment Map files, a compression of the sequence that can be annotated to explain particular details; Variant Call Format (VCF) files, providing information on the type, number, and position of nucleotide variants; single-nucleotide polymorphism (SNP) files detailing changes in single base pairs in the DNA sequence; risk score files, which contain information on risks of health conditions based on single or multiple genes; and among the simplest are files that detail the presence or absence of a trait of interest (Daniels H et al, unpublished data, 2019). Using genomic information in conjunction with additional data can add valuable detail and provide context in health conditions. However, importantly, the relative risks will differ depending on the form of genomic data being used, along with other factors. We take this into account in the development of the recommendations.

### Examples Using Genomic and Phenotypic Data

We provide examples of studies using various forms of genomic data and phenotypic data to illustrate data use for research with a view to considering relative risks. As well as GWAS and PheWAS studies that require the full genome sequence for large-scale statistical analysis to identify variants of interest, many studies begin with sequence but subject it to further processing to create derivatives for linkage to structured phenotypic data. For example, polygenic risk scores derived from GWAS and SNPs then linked to an index of deprivation and individual postcodes to explore factors influencing alcohol dependence [23]. Genotyping at particular loci has been used with the number and duration of in-patient events in schizophrenia [24]. Gene expression profiling has been used in conjunction with electronic health records (EHRs) to compare breast cancer treatments and predict chemotherapy efficacy and outcomes across health care systems [25].

SNPs are among the forms of genomic data commonly used in conjunction with phenotypic data. These were used in a study on major depressive disease with linkage to health service data, including diagnoses, history of antidepressant prescribing, and referrals to secondary care for specialist treatment [26]. A study on dementia used a set of 6 SNPs linked to EHRs to establish and monitor the dementia status of participants [27]. A further example is the use of SNPs to study herpes zoster linked to EHRs to determine diagnosis in adults [28]. EHRs are often the source of phenotypic data, but information may also be drawn from registries [29,30], and in some cases, area-based measures, such as indices of deprivation, are included [27].

As for any studies using individual-level health data, risks will vary depending on the specifics of the use case. In relation to genomic data with phenotypic data, variables that may influence risk include forms of genomic data, common versus rare conditions, studying sensitive or stigmatizing conditions, and the extent of data linkage to phenotypic data. These are in addition to demographic factors such as extremes of age and small geographies, and all may combine to create the risk profile. As such, the degree of disclosure risk varies, but the repercussions of reidentification also vary. For example, the unauthorized disclosure that someone’s close blood relative carries a high risk of developing a strongly hereditary condition such as Huntington disease will have serious implications for themselves and their kin. On the other hand, conditions such as heart disease and diabetes are multifactorial and, as such, are more complex and indefinite in terms of disease prediction. These and a myriad of other considerations lead us to propose that the risks and benefits of planned data uses should be taken into account on a case-by-case basis.

### Data Governance Arrangements

We used information from the literature review and the series of interviews with representatives of safe havens on data governance arrangements in place. We refer only to governance arrangements in relation to the use of genomic data, rather than the work of these enterprises in general. We found that differing solutions have been put in place to enable genomic data to be used in conjunction with phenotypic data. Examples are given here to show variety.

The German Medical Informatics Initiative is a major infrastructural investment that has created multiple university hospital consortia to integrate clinical data, including genomics [31]. In terms of genomic data, the work of this new initiative is beginning with biosample collection before the results of genomic analyses will be shared across consortia. Germany is subject to the GDPR but also has stringent national privacy regimens for general and genomic data processing [32,33]. Moreover, apart from repositories holding data locally, methods for cross-center distributed analyses, such as DataSHIELD, are being employed [34]. These will bring the analysis to the data and avoid the need to share data where this is deemed unacceptable to oversight committees. Subject to approval, data can be used within consortia, across consortia, and by any approved researcher. However, as noted, this might not involve direct access to data but might depend on privacy-preserving analysis methods. There is a network of committees and working groups for the establishment of agreed standards.

The Sax Institute in Australia manages the *45 and up* study, a longitudinal cohort of over 250,000 people, including phenotypic and genomic data [35]. Sax works in partnership with the Garvan Institute of Medical Research, which acts as a genome sequencing facility and makes data available for research subject to conditions [36]. Sax operates as a repository for health and self-report data, whereas Garvan acts as a repository for the genomic data. Although the full genome data are generally too large to move, other data such as VCF files could be transported; thus, the decision to use a distributed model is based on data governance reasons. Researchers need to apply to both institutional data access committees before access to genomic and phenotypic data can be granted. Linked data can be accessed by public and private sector researchers via a portal subject to ethical and other relevant approvals. Following analysis, outcomes are released externally but not row-level data.

The UK Secure Research Platform (SeRP) is a data infrastructure housing the Secure Anonymised Information Linkage (SAIL) databank and various other initiatives [15,37]. UK SeRP can be customized to implement the data governance model required by particular programs of work. To date, SAIL has integrated genomic data on a project basis but is working to incorporate more genomic data as part of standard data feeds, along with phenotypic data from health and administrative records. Example projects involve a psychosis cohort and an epilepsy study. As the genomic data were brought into SAIL for particular projects, separate ethical approval including participant informed consent was obtained. The psychosis cohort brought in polygenic risk scores and copy number variants; the epilepsy study brought in VCF files. In both cases, the data were only made available to project researchers because of regulatory approvals. But in general, SAIL allows data access to any approved public sector researcher; the commercial sector must work with a SAIL analyst or another public organization to access data on their behalf. All proposals to access data for research must be approved by an information governance review panel that co-opts additional experts in assessing particular data types, such as genomic. Some data providers also reserve the right to review data use proposals, in addition to the panel.

The Institute for Clinical Evaluative Sciences (IC/ES) operates in Ontario, Canada [38], and holds a highly phenotyped cohort of over 2000 children and young people with a neurological development disorder, most frequently, autism. DNA samples were collected for whole genome sequencing from the entire cohort, and the ones who consented to linkage have their VCF file data linked to IC/ES health and administrative data. As such, this is a project-level development, but it is anticipated that linkage of genomic data to phenotypic data will become more routine in future. The privacy approval group at IC/ES was concerned about identifiability because the genomic data are often unique, particularly where there are rare variants. Owing to this, the arrangement at IC/ES has been to hold the genomic data separately and not on the main analysis platform. As the linked data are considered highly sensitive, only an IC/ES analyst has access. The project lead prepares queries to be executed on the data and receives the results from the IC/ES analyst.

From these examples, it can be seen that data safe havens are at different stages of development in terms of integrating genomic data, they are working with various formats of genomic data, and they have varying perceptions of risk leading to differing requirements for data access. These are just some examples, with others underway such as the Swiss Personalized Health Network and its associated BioMedIT project. Through this, there is a network of core facilities for the secure processing of biomedical data across Swiss universities, enabling research within a secure environment [39]. However, although models differ, one of the common beauties of safe havens (over open access or external release of data) is that they are able to apply a suite of disclosure controls directly to the data (in totality or at a project level) and across the whole environment, thus managing risk across all stages from data incorporation to archiving [14,15,38]. We used the information gained from these various models to guide the development of the recommendations.

### Public Views

Having previously reported public perspectives on 3 models of accessing genomic data [11], here we draw upon the views gained on data use in safe havens, in line with the focus of this paper. In general, workshop participants were less concerned about the use of genomic data in safe havens than external release or open access. However, there were some provisos in relation to safe havens. Participants wanted to be properly informed on the purpose of data use and for analyses to be conducted by approved researchers, with concern expressed on misuse by commercial companies. They wanted to be reassured that appropriate safeguards would be in place, with data use being auditable and controlled. The need for consent to reuse genomic data that had been primarily collected for research emerged strongly (for all access models) and led us to propose wording for the information sheet and consent form in prospective studies [11]. The use of anonymized data in safe havens was also seen as a way to mitigate risks of discrimination because access is limitable by systematic controls. The public viewpoints on the use of genomic data in safe havens guided us in the creation of recommendations.

## Discussion

### Principal Findings

Having considered the legislative backdrop in the EU, it can be seen that there is nothing inherently different in the EU GDPR about the lawful provisions for genomic data processing compared with other health data: all are classed as special category data. However, there is much debate about the effectiveness of anonymization processes for genomic data, and some consider that it is particularly difficult to produce genomic data that are both anonymous and useful [3,10]. This is not a new thought as it has long been noted in relation to demographic and health data [40], but it has been specifically applied because of the nature of genomic data leading to the concept of genetic exceptionalism [6]. Some of this is because of factors such as persistence, discrimination, and impact on kin, but there is an apparent mystique around genomic data that is proving challenging even if it is nonempirical, as beliefs play a significant role in policy making. The perceived complexities surrounding genomic material and data can lead to an overly cautious and proscriptive approach to the detriment of research [3]. We fully acknowledge that there are risks to be mitigated for safe data use and that data misuse can cause harm to individuals and professional reputations. However, it is also true that serious harm occurs because of data nonuse, far more than the missed benefits of proper data usage and leading to hundreds of thousands of lost lives and billions of dollars in financial burdens to societies [41]. In fact, it has been postulated that nonuse is often a greater problem than unauthorized data use [42].

A particularly important issue is the problem of conflating unique and identifiable. It cannot be overstressed that just because data are unique, this is not the same as being identifiable *per se*. If research had to rely on using only the data present in equivalence classes (ie, where there are no unique records, only sets of records [minimally 2] with identical variables), progress would be hampered. Even the fact that a person’s full genome is unique does not render it identifiable without considerable effort, interpretation, and additional information to confirm identity. This has been shown in the debunking of a study purporting to identify individuals from genomic data, revealing that the work only narrowed down the information to a category of people based on phenotypic traits [43,44]. That is not to say that new insights on current limited knowledge of the genome will not change the likelihood of identifiability. Future proofing is important in the use of all personal data but more so in relation to genomics where successive new understandings are being revealed from previously unknown data content as the genomic revolution advances apace.

Another important factor to consider is what is meant by genomic data when special processing rules are suggested. As we have seen from the literature review and case study interviews, genomic data are used in various forms along with a variety of phenotypic data. Although GWAS and PheWAS studies rely on DNA sequence, many other studies use only DNA derivatives, or metadata, of varying complexity. We propose that it is not appropriate to bundle genomic data into one category and assume a uniformly high level of privacy risk. Even so, there is more than legislation to consider when seeking to use personal data for research, including ethical and social implications. Among the safe havens currently working with genomic data, there is a variety of approaches to data governance in accordance with their jurisdictional frameworks and interpretations. Depending on how the genomic data were collected, there may be a requirement for informed consent or data may be incorporated via standardized feeds along with health and administrative records based on sharing agreements with data providers. Data access might be limited to institutional employees or the public sector or might be open to all sectors. Researchers might be provided with direct access to linked data or to be limited to distributed queries. Genomic data are sometimes stored with the phenotypic data, but they might be on separate platforms and are sometimes held by separate organizations with access provided on a distributed model or with only limited derivative data imported. Commonly, safe havens do not generally release row-level data but export products of analysis following disclosure risk scrutiny. In terms of social acceptability, at least from our previous work, the public were generally favorable toward the use of genomic data in safe havens, subject to provisos including privacy safeguards and being provided with information about data uses.

### Recommendations

We use the findings of the various elements of this study to propose a set of recommendations toward creating a risk-utility data governance framework and to augment existing operating models for using genomic and phenotypic data for research in an anonymized form in safe havens. We present the recommendations as overarching principles and practical control measures to mitigate risks and retain maximum data utility.

#### Principles

We propose that these principles be applied in general as part of the data governance framework for safe havens incorporating genomic data. We align the principles with the core elements presented in the high-level framework for the use of genomic and health data developed by the GA4GH [20], with the main relevant elements shown in italic text. The principles are as follows:

- Jurisdictional data protection legislation for general data processing, any specific provisions for genomic data processing, and relevant authoritative guidance and codes of practice should be examined and properly interpreted, with the input of legal expertise to ensure due compliance, *transparency,* and *accountability.*
- The need for regulatory approvals (including research ethics and informed consent) and data provider permissions for incorporating genomic data should be assessed, and all due diligence should be followed to demonstrate *transparency* and *accountability* and to support *recognition* and *attribution* in data provision.
- Members of the public should be provided with the opportunity to be properly *engaged* and involved at the strategic level to contribute their views to informing developments for the systematic incorporation of genomic data. The respectful need for a measure of *education and training* and the *dissemination* of information in *accessible* forms should be accommodated for transparency and accountability to the public, from whom the data arise.
- There is a need for a flexible, proportionate approach to consider proposed uses of genomic data, as they come in many formats with varying privacy risks, and (at least at present) it is incorrect to assume that genomic data are identifiable simply by reason of being unique. Assessment should be made on a case-by-case basis using *risk-benefit analysis* and with *education and training* to support data access committees.
- Safe havens should use their combination of data environment, physical, technical, and procedural controls, coupled with disclosure controls applied directly to the data, to maximize *data quality and security, privacy,* and *data protection and confidentiality.*
- As knowledge in the field of genomics is advancing rapidly, it is especially important to ensure future proofing of individual *privacy, data protection, and confidentiality* of genomic information. For *accountability* and *sustainability* of data use, we recommend that the practice of only exporting products of analysis should be the general policy, particularly for linked genomic and phenotypic data. Exceptions could be made if all relevant approvals are in place and the data are to be moved to another safe haven.

#### Practical Control Measures

The following is a selection of practical control measures that can be applied to specific cases where genomic and phenotypic data are used in combination to mitigate risk and retain maximum utility. They are not sequential or hierarchical but can be used in various combinations. The proposed control measures are as follows:

- All relevant project-level regulatory approvals for the collection and processing of genomic data should be checked by the data access committee, as part of their assessment of data use suitability.
- Researchers should consider opportunities for public engagement on their proposed use of genomic data to gain input on direction and preliminary findings.
- Along with the standing membership of the data access committee, a genomics expert should be co-opted to review particular proposals.
- Case-by-case review should take into account factors including the form and extent of genomic data to be used and the rarity and sensitivity of health and lifestyle factors to be studied, in addition to the criteria generally used by the data access committee.
- Reviews should include who will be permitted to access the data, depending on perceived risk and regulatory and data provider requirements. If deemed necessary, data access should be limited to the project team, a single researcher, or only to an analyst employed by the safe haven host, tasked with producing results to share with the project team.
- Many safe havens provide remote data access for approved researchers at their desktop, wherever they are based. If deemed necessary, data access should be physically restricted to a designated safe room so that a researcher has to be present on site when analyzing the data, and data access should be subject to stringent monitoring.
- Data granularity can be curtailed using a variety of disclosure control techniques applied to the data. The choice of methods should be selected with care in discussion with the researcher to retain maximum utility and safeguard the data.
- If available to the safe haven, the option of using distributed queries where the researcher is not provided with a view of the data but sends their query to the server and receives results should be considered for proposals deemed particularly risky.
- Where genomic data cannot be moved from the source, a hybrid model should be considered. Instead of incorporating the genomic data into the data safe haven, a federated access model may be used. However, the feasibility of this will depend on available technology and resources.

From our findings we anticipate that, in many cases, genomic data can be used safely and appropriately in conjunction with phenotypic data within a safe haven without many (if any) major changes to the current operating models, making use of the suite of controls available. The use of various forms of genomic data with phenotypic data will often be unlikely to present significant additional risks over and above those posed by the use of multiple linked health and administrative records within the safe haven. Provided that a case-by-case assessment is made, and proportionate controls are applied to mitigate risks while transparently acknowledging that they might not be totally eradicated, there should be no reason for not permitting the use of genomic data for research in safe havens.

### What This Study Adds

This is the first known study to propose recommendations toward a risk-utility data governance framework for the use of genomic and phenotypic data in safe havens. It has brought together findings from published research, case studies of data safe havens, and public views against a backdrop of (EU) data protection legislation to inform the perspectives presented. It is a novel, evidence-based study that can be used to guide existing and newly developing data safe havens on working with genomic data to safeguard the data without falling into the trap of privacy protectionism [7], but still ensuring risks are properly mitigated while retaining maximum data utility. We do not claim to have solved all the challenges or that risk can be totally eradicated, but the study has shed new light on routes toward a risk-benefit data governance framework to use genomic and phenotypic data safely and effectively.

### Limitations

We acknowledge limitations to this study. It is based on a nonexhaustive literature review, views of a limited number of people based on a variety of settings in Wales, case studies of some data safe havens, and the main EU data protection legislation. It is possible that various other pieces of information pertaining to other jurisdictions, organizations, and cultures may differ. However, we are not proposing that our findings are ultimately definitive but that they can be used toward a data governance framework, taking necessary differences into account.

### Conclusions

This study acknowledges the benefits and challenges in using genomic data in conjunction with phenotypic data and the need for guidance to promote the safe, socially acceptable use of data in data safe havens. We used a multifaceted approach to propose evidence-based recommendations toward a risk-utility data governance framework based on a suite of controls applied to and around the data to mitigate risks and retain data utility. They do not purport to eradicate risk but propose case-by-case assessment with transparency and accountability. If the risks are adequately understood and mitigated, there should be no reason that linked genomic and phenotypic data should not be used in an anonymized form for research in safe havens.

## Abbreviations

EHR: electronic health record
EU: European Union
GA4GH: Global Alliance for Genomics and Health
GDPR: General Data Protection Regulation
GWAS: genome-wide association study
IC/ES: Institute for Clinical Evaluative Sciences
PheWAS: phenome-wide association study
SAIL: Secure Anonymised Information Linkage
SeRP: Secure Research Platform
SNP: single-nucleotide polymorphism
VCF: Variant Call Format
